# Imbalance in Renal Vasoactive Enzymes Induced by Mild Hypoxia: Angiotensin-Converting Enzyme Increases While Neutral Endopeptidase Decreases

**DOI:** 10.3389/fphys.2018.01791

**Published:** 2018-12-11

**Authors:** Carlos P. Vio, Daniela Salas, Carlos Cespedes, Jessica Diaz-Elizondo, Natalia Mendez, Julio Alcayaga, Rodrigo Iturriaga

**Affiliations:** ^1^Department of Physiology, Center for Aging and Regeneration CARE UC, Facultad de Ciencias Biológicas, Pontificia Universidad Católica de Chile, Santiago, Chile; ^2^Facultad de Medicina y Ciencia, Universidad San Sebastián, Santiago, Chile; ^3^Institute of Anatomy, Histology, and Pathology, Facultad de Medicina, Universidad Austral de Chile, Valdivia, Chile; ^4^Laboratorio de Fisiología Celular, Departamento de Biología, Facultad de Ciencias, Universidad de Chile, Santiago, Chile; ^5^Laboratorio de Neurobiología, Department of Physiology, Facultad de Ciencias Biológicas, Pontificia Universidad Católica de Chile, Santiago, Chile

**Keywords:** renal hypoxia, kallikrein, angiotensin-converting enzyme, neprilysin, neutral endopeptidase, subtle renal injury

## Abstract

Chronic hypoxia has been postulated as one of the mechanisms involved in salt-sensitive hypertension and chronic kidney disease (CKD). Kidneys have a critical role in the regulation of arterial blood pressure through vasoactive systems, such as the renin-angiotensin and the kallikrein–kinin systems, with the angiotensin-converting enzyme (ACE) and kallikrein being two of the main enzymes that produce angiotensin II and bradykinin, respectively. Neutral endopeptidase 24.11 or neprilysin is another enzyme that among its functions degrade vasoactive peptides including angiotensin II and bradykinin, and generate angiotensin 1–7. On the other hand, the kidneys are vulnerable to hypoxic injury due to the active electrolyte transportation that requires a high oxygen consumption; however, the oxygen supply is limited in the medullary regions for anatomical reasons. With the hypothesis that the chronic reduction of oxygen under normobaric conditions would impact renal vasoactive enzyme components and, therefore; alter the normal balance of the vasoactive systems, we exposed male Sprague-Dawley rats to normobaric hypoxia (10% O_2_) for 2 weeks. We then processed renal tissue to identify the expression and distribution of kallikrein, ACE and neutral endopeptidase 24.11 as well as markers of kidney damage. We found that chronic hypoxia produced focal damage in the kidney, mainly in the cortico-medullary region, and increased the expression of osteopontin. Moreover, we observed an increase of ACE protein in the brush border of proximal tubules at the outer medullary region, with increased mRNA levels. Kallikrein abundance did not change significantly with hypoxia, but a tendency toward reduction was observed at protein and mRNA levels. Neutral endopeptidase 24.11 was localized in proximal tubules, and was abundantly expressed under normoxic conditions, which markedly decreased both at protein and mRNA levels with chronic hypoxia. Taken together, our results suggest that chronic hypoxia produces focal kidney damage along with an imbalance of key components of the renal vasoactive system, which could be the initial steps for a long-term contribution to salt-sensitive hypertension and CKD.

## Introduction

Hypertension and chronic kidney disease are major public health problems worldwide, with chronic hypoxia being one of the suggested mechanisms involved in the genesis of salt-sensitive hypertension and to the progression of CKD (Suga et al., 2001; Nangaku and Fujita, 2008). Several factors, such as angiotensin II, cyclosporine A, phenylephrine, and hypokalemia have been postulated to induce local vasoconstriction and renal hypoxia, which subsequently may lead to CKD (Johnson et al., 2002; Vio and Jeanneret, 2003).

Renal function and arterial blood pressure regulation are under physiological control by renal vasoactive substances, through the activation of different systems such as the following: nitric oxide synthases, the endothelin system, the renin-angiotensin system (RAS), the kallikrein–kinin system (KKS), and renal eicosanoids produced by cyclooxygenases 1 and 2. Among nitric oxide synthases, neuronal nitric oxide synthase (nNOS, NOS-1) plays an important role in the kidneys by regulating renal hemodynamics in the immature kidney during pre and postnatal stages (Rodebaugh et al., 2012). Additionally, NOS-1 and NOS-3 are upregulated by acute hypoxia (Gess et al., 1997). On the other hand, endothelin-1, which is a multifunctional peptide, has potent vasoconstrictor and profibrotic effects on the systemic vasculature and kidneys. The endothelin system seems to play a fundamental role in diabetes, proteinuric renal disease, hypertension, and renovascular disease (Hargrove et al., 2000; Kelsen et al., 2011; Meyers and Sethna, 2013). Our laboratory mainly studied the following two systems: the RAS, the KKS, and renal eicosanoids, which are related to both systems. We have focused on enzymes in both systems, since they are currently known as important targets of inhibitors in antihypertensive treatment, and are used in CKD. The RAS leads to vasoconstriction and sodium retention, and its main active peptide is angiotensin II. The latter is produced by the angiotensin-converting enzyme (ACE), and is metabolized by aminopeptidase A to angiotensin III (Mizutani et al., 2008) and by neutral endopeptidase 24.11 (NEP) or neprilysin to inactive metabolites (Rice et al., 2004; Campbell, 2017). The KKS produces vasorelaxation and sodium excretion through bradykinin produced by kallikrein, which is the key enzyme of the system. The renal KKS participates in renal and extrarenal events such as regulation of blood pressure and control of sodium and water excretion. Kallikrein originates in the connecting tubule (CNT) and generates bradykinin, which is the effector hormone in the kidney regulating sodium excretion and glomerular hemodynamics, among other effects (Jaffa et al., 1992; Vio et al., 1992; Madeddu et al., 2007; Zhang et al., 2018). Bradykinin levels are regulated by ACE and NEP. There is another family of natriuretic peptides that participates in the regulation of arterial blood pressure, with atrial natriuretic peptide being the main one that involves renal action (Chen, 2005). Furthermore, the availability of vasoactive hormones is regulated by peptidases that degrade them. In kidneys, NEP has the critical function of degrading bradykinin, angiotensin II and natriuretic peptides, and transforming angiotensin I into angiotensin 1–7 (Rice et al., 2004; Judge et al., 2015; Campbell, 2017).

Renal vasoactive systems are involved in a delicate balance of opposite effects that lead to the production of either vasoconstrictor or vasodilator hormones, which activate sodium-retaining or excretory mechanisms, and have profibrotic or antifibrotic consequences (Bader and Ganten, 2008). For example, ACE and NEP have opposite actions in the regulation of vasoactive peptides: ACE transforms angiotensin I into angiotensin II (with hypertensive effects) and NEP transforms angiotensin I into angiotensin 1–7 (with antihypertensive effects). Angiotensin 1–7 can be further degraded by ACE into angiotensin 1–5 (Rice et al., 2004; Campbell, 2017).

Alterations of vasoactive enzymes have been described during the progression of CKD and hypertension. In fact, most drugs used to treat hypertension have been designed to inhibit members of the RAS family. Furthermore, new studies suggest that the use of the angiotensin II receptor antagonist in addition to the NEP inhibitor is a promising strategy to treat heart failure associated to hypertension (Volpe et al., 2016).

It is known that the kidney is very sensitive to changes in oxygen supply. Partial pressure of oxygen (PO_2_) is carefully balanced between the cortex and the outer medulla, where under normoxia, the PO_2_ gradient within the kidney has been found to reach about 70 mmHg in the cortex and outer medulla, and up to 10 mmHg in the inner medulla and papilla (Leichtweiss et al., 1969; Baumgärtl et al., 1972; Günther et al., 1974; Lübbers and Baumgärtl, 1997; O’Neill et al., 2015). Adequate kidney oxygenation is crucial to fuel active transportation processes of electrolytes and water in the nephron. Although kidneys receive a very high blood flow, oxygen extraction is relatively low. Consequently, kidneys are particularly susceptible to hypoxic injury because small changes in flow, which can be generated by vasoconstriction for example, are translated into local hypoxia (Epstein, 1997). This can then generate focal lesions, and lead to manifestations of kidney damage when the condition becomes chronic.

Based on the current knowledge exposed above, the present study was guided by the hypothesis that chronic reduction of oxygen, under normobaric conditions, affects renal vasoactive enzyme components, altering the normal balance of vasoactive systems, and favoring the vasoconstrictor profibrotic RAS.

## Materials and Methods

### Animals and Experimental Procedures

Experiments were performed in 12 adult male Sprague-Dawley rats (180–200 g, *n* = 6 for each group, normoxia and hypoxia). This study was carried out in accordance with recommendations in the “Manual de Normas de Bioseguridad” (*Biosafety Norms Manual*, 2nd Ed., 2008, FONDECYT-CONICYT). The experimental protocol for animals was approved by the Bio-Ethics Committee of the School of Science at Universidad de Chile. Animals were housed in a 12 h light/dark cycle with free access to food (Prolab RMH 3000, Purina LabDiet) and water, and were randomly assigned to either a control group (normoxia) or to chronic normobaric hypoxia for 2 weeks.

### Chronic Normobaric Hypoxia Exposure

Animals (*n* = 12) were exposed to normoxia (*n* = 6), serving as controls for normobaric hypoxia (*n* = 6) for 2 weeks as previously described (Icekson et al., 2013). Briefly, three rats were allocated to a cage (D × W × H; 48 cm × 26 cm × 15 cm) and two cages were placed in a 300 L (60 cm × 50 cm × 100 cm) acrylic chamber with a hermetic lid. The oxygen content (FiO_2_) in the chamber was continuously monitored through an oxygen sensor (AX300, Teledyne Analytical Instruments, CA, United States), whose output was fed to an automatic programmable controller (Zelio SR2B121BD, Schneider Electric, France) that controlled two solenoid valves (2026BV172, Jefferson Solenoid Valves, FL, United States). A relief output valve opened simultaneously with the admission valves, and remained opened for 40 s after the closure of the latter ones, while two mechanic relief valves opened whenever the pressure inside the chamber exceeded approximately 17 mmHg. Thus, the mean pressure in the chamber was slightly larger (ΔP = 1.3 ± 0.1 mmHg) than the atmospheric one (717.2 ± 0.3 mmHg). The atmosphere within the chamber was continuously homogenized by four internal fans. After closure, chamber air was purged for approximately 5 min with pure N_2_, until attaining approximately 9.5% of FiO_2_. From then on, the system automatically regulated FiO_2_ levels in the chamber by admitting N_2_ or compressed air into the chamber if FiO_2_ values were over 9.8% or below 8.7%, respectively. Mean FiO_2_ within the chamber was 9.33 ± 0.12% (mean ± SD). CO_2_ produced by animal ventilation was trapped by using CaCO_3_ (250 g), and urinary NH_3_ with H_3_BO_3_ (60 g). The chamber remained open for about 5 min every other day to clean the internal cages and replenish the water containers, whereas CO_2_ and NH_3_ traps were changed every four days. The cages were rotated within the chamber to maximize homogeneity. The pressure inside the chamber was continuously measured with a gauge transducer (Statham P20), and the FiO_2_ signal was recorded at 1 Hz with a computerized analog to digital acquisition system (DI-158U, DATAQ Instruments Inc., OH, United States). The temperature in the chamber was recorded at 5 min intervals throughout the 14 days of hypoxia with a data logger (EL-USB-2, Lascar Electronics Inc., PA, United States).

At the end of the treatment, rats were deeply anesthetized with isoflurane (isoflurane in O_2_, induction 4–5%) and euthanized by exsanguination. Blood was obtained from Vena Cava in heparin-tubes, and both kidneys were removed. Then, isoflurane was increased until breathing stopped and death of the animals was confirmed. Pneumothorax was performed as a secondary physical method of euthanasia.

Kidneys were rapidly decapsulated and approximately 100 mg slices were cut with a transverse cross section in the middle of the kidney (discarding both poles) to obtain samples for quantitative polymerase chain reaction (qRT-PCR). Samples for qRT-PCR were stored at –80°C until they were processed.

The hematocrit was determined by microcentrifugation using two uncoated hematocrit glass tubes (length 75 mm) per sample, filled and centrifuged for 5 min in a micro-hematocrit centrifuge (Hermle Z200). The hematocrit was established using a micro-hematocrit tube reader, and the mean value was calculated.

### Source of Antisera and Chemicals

ACE antiserum (SC-12187) was purchased from Santa Cruz Biotechnology, United States. Osteopontin (OPN) monoclonal antibody (MPIIIB10) was obtained from Developmental Studies Hybridoma Bank, IA, United States, and NEP antiserum (AB-5458) from EMD Millipore. Kallikrein antibody was used as reported previously (Salas et al., 2007). Random primer dNTPs and FAST SYBR Green Master Mix were acquired from Thermo Fisher Scientific. Secondary antibody and corresponding peroxidase-anti-peroxidase (PAP) complex were purchased from MP Biomedicals, Inc., Germany.

### Tissue Processing and Immunohistochemical Analysis

Renal tissue samples (3 mm thick) were fixed by immersion in Bouin’s solution for 24 h at room temperature. Tissue was then dehydrated, embedded in Paraplast Plus, serially sectioned at 5 μm thick with a Leica rotatory microtome, mounted on glass slides, and stored.

Conventional staining of tissue sections was performed with Hematoxylin-Eosin (H-E), Periodic Acid-Schiff (PAS) for histological analysis, and Picrosirius red for collagen staining (Villanueva et al., 2006, 2014).

Immunostaining was carried out using an indirect immunoperoxidase technique to localize kallikrein, ACE, OPN, and NEP in rat kidneys. Briefly, tissue sections were dewaxed, rehydrated, rinsed in 0.05 M Tris-phosphate-saline (TPS) buffer, pH 7.6, and incubated overnight at 22°C with a primary antiserum raised against kallikrein, ACE, OPN, or NEP. The secondary antibody and corresponding PAP complex were applied for 30 min each at 22°C. The immunoperoxidase reaction was visualized after incubation of sections in 0.1% (wt/vol) diaminobenzidine and 0.03% hydrogen peroxide. Sections were rinsed with TPS buffer between incubations, counterstained with hematoxylin, dehydrated, cleared with xylene and then coverslipped. Controls for the immunostaining procedure were prepared by omission of the first antibody. Images were examined with conventional light microscopy and acquired using a Nikon Eclipse E600 microscope and Nikon DS-Ri1 digital camera.

### Quantitative RT-PCR

Total RNA was extracted from whole kidney tissue using TRIzol, according to the manufacturer’s instructions. RNA integrity was determined by 1% agarose gel electrophoresis and its concentration, by absorbance at 260/280 nm using a 2.5 μg aliquot of total RNA to cDNA synthesis. Samples were treated with DNAse I using MMLV, dNTPs and random primers to obtain cDNA. The housekeeping gene used was glyceraldehyde-3-phosphate dehydrogenase (GAPDH) (forward primer: 5′-CACGGCAAGTTCAACGGC-3′, reverse primer 5′-GGTGGTGAAGACGCCAGTA-3′). The primer sequences used were the following: kallikrein, forward primer: 5′-GCATCACACCTGACGGATTG-3′, reverse primer: 5′-GGCCTCCTGAGTCACCCTTG -3′; ACE, forward primer: 5′-AACACGGCTCGTGCAGAAG-3′, reverse primer: 5′-CCTGCTGTGGTTCCAGGTACA-3′; and NEP, forward primer: 5′-TCAGCCTTTCTGTGCTCGTC-3′, reverse primer: 5′-ATTGCGTTTCAACCAGCCTC-3′. Quantitative PCR was performed in duplicate in a StepOnePlus Real-Time PCR System (Applied Biosystems) using FAST SYBR Green Master Mix for amplification. Results were normalized by GAPDH. Mathematical quantification was made using the 2^-ΔΔCT^ method (Livak and Schmittgen, 2001).

### Statistics

All data are presented as mean ± SEM. Statistical analyses were performed using an unpaired Student’s *t*-test and GraphPad Prism software (version 5.0c for Windows, GraphPad Software, CA, United States). Differences with *P* < 0.05 were considered statistically significant.

## Results

### General Features and Morphological Traits

An increase in hematocrit and a decrease in body weight were observed in hypoxic animals compared to controls, over the 2-week period of hypoxia, which is characteristic of this experimental condition (Table 1).

**Table 1:** Body weight and hematocrit values in normoxic and hypoxic animals.

	Normoxia	Hypoxia
Body weight (g)	337.8 ± 7.7	219.3 ± 5.2^∗^
Hematocrit (%)	44.3 ± 0.3	74.2 ± 1.2^∗^

*^∗^*P* < 0.001*.

For the morphological study we selected conventional stains with different properties to address tissue structure at the light microscopy level. They were H-E, PAS and Picrosirius Red. They provide information about general structure (H-E), more detailed information of structural shapes of tubules, glomeruli and vessels, provided by the glycoprotein and basement membrane staining (PAS), and collagen staining as an index of tissue damage (Picrosirius red).

The immunohistochemistry technique was used to evaluate *de novo* expression of tubular OPN, which is a key macrophage chemokine that is not normally expressed in adult kidneys, and is induced by tubular damage of different origins (Lombardi et al., 2001).

Also, immunohistochemistry was used to characterize the protein expression at tissue level and the distribution in the kidney of the vasoactive enzymes kallikrein, ACE, and NEP.

Renal tissue specimens were examined in coded samples by two of the researchers (C.Cespedes and C.Vio), and systematic analysis of kidney tissue samples was done focusing on the overall morphological aspect, and on cortical and medullary tubules, glomeruli, blood vessels, interstitial space, and intratubular spaces.

**FIGURE 1:**
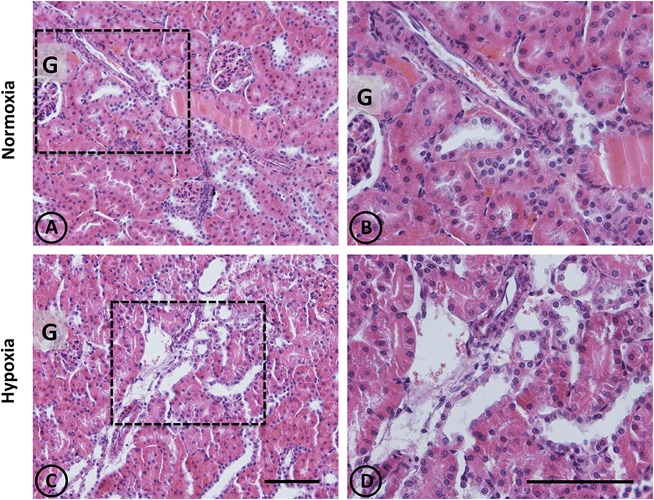
Conventional staining with H-E in renal tissue from normoxic and hypoxic animals. Representative images of kidneys from animals in control **(A,B)** and hypoxic **(C,D)** conditions. **(A,B)** Normoxic tissue has tubules and tubulointerstitial space of normal aspect, both in the medullary zone and in the cortex. Blood vessels present usual appearance. **(C,D)** In hypoxic kidneys, it is possible to observe tubular dilation in some areas of the field, affecting certain blood vessels that irrigate such areas. Dotted areas correspond to higher magnification images on the right column. G = Glomerulus. Scale bar = 100 μm.

**FIGURE 2:**
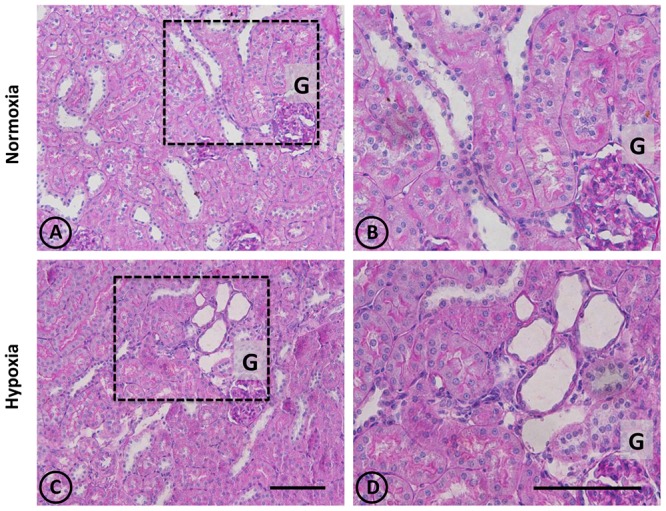
Conventional staining with PAS in renal tissue from normoxic and hypoxic animals. **(A,C)** show a general view of cortical and medullary zones for each group. **(A,B)** correspond to the normoxia group. The color fuchsia is present mainly in the basal membrane and the brush border of proximal tubules. No signs of injury are observed in this group. **(C,D)** correspond to the hypoxia group, showing an area that presents focal damage. Only a few tubules present dilation, while the area that surrounds it presents normal appearance. Dotted areas indicate higher magnification images presented in the right column. G = Glomerulus. Scale bar = 100 μm.

An overall and detailed examination of renal tissue with conventional PAS and H-E staining of kidneys from normoxic animals showed no signs of pathological alterations in the cortex or the medulla. No vascular changes were observed, tubules were shaped normally in terms of diameter and cell size, and no signs of cell infiltration and inflammation were observed in the tubulointerstitial space. Glomeruli from cortical or juxtamedullary nephrons had normal aspect (Figures 1A,B, 2A,B).

A panoramic examination of kidney tissue from hypoxic animals over the cortex and medulla revealed a mild and focalized morphological alteration, characterized by focal lesions in the outer medullary and inner cortical zone, as evidenced with H-E staining (Figure 1). More detailed information was obtained with PAS staining, showing the focal alteration at this outer medullary region (Figure 2).

Focal areas with signs of alterations consisted of “spotty” areas mainly located in the outer medulla and inner cortical zone, close to juxtamedullary nephrons (Figures 1, 2). They contained dilated tubules with atrophic tubular epithelia, and basophilic aspect. Such atrophic and dilated tubules had a mild cellular infiltration in the corresponding tubulointerstitial space (Figures 2C,D).

**FIGURE 3:**
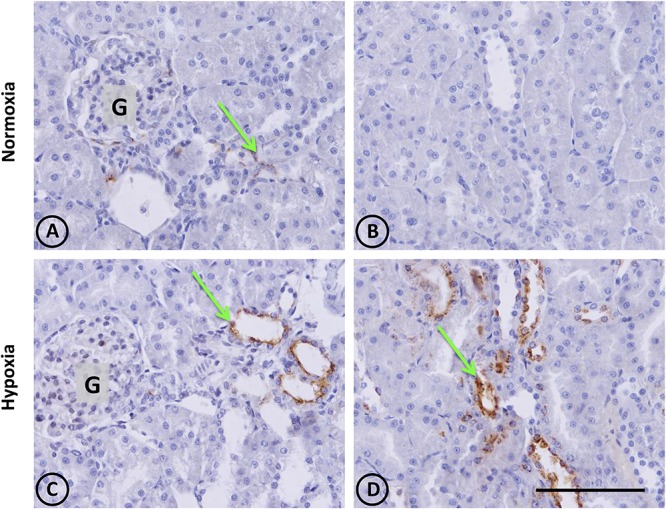
Osteopontin in renal tissue from normoxic and hypoxic animals. The arrows indicate the localization of OPN that appears stained in brown. **(A,B)** correspond to the normoxia group. In **(A)** it is possible to observe a little OPN stain that indicates low expression levels of this protein. Brown stains are totally absent in **(B)**, and the same happens in others fields of the same renal section, representing normal conditions. **(C,D)** correspond to the hypoxia group. It is possible to observe an increase of OPN staining, expressed mainly in tubular cells. The left panel shows a cortical area where a glomerulus is observed (G). The right panel corresponds to the outer stripe of the outer medulla. All images have the same magnification. Scale bar = 100 μm.

In control animals, the presence of renal OPN was observed with a very scarce distribution in tubular cells of the cortex, as expected for normal tissue (Figures 3A,B). In contrast to normal tissue, kidneys from hypoxic animals showed an increased expression of OPN in cortical tubules with a focal distribution, as shown in Figures 3C,D, which corresponds to the typical pattern of *de novo* expression of OPN in renal injury.

**FIGURE 4:**
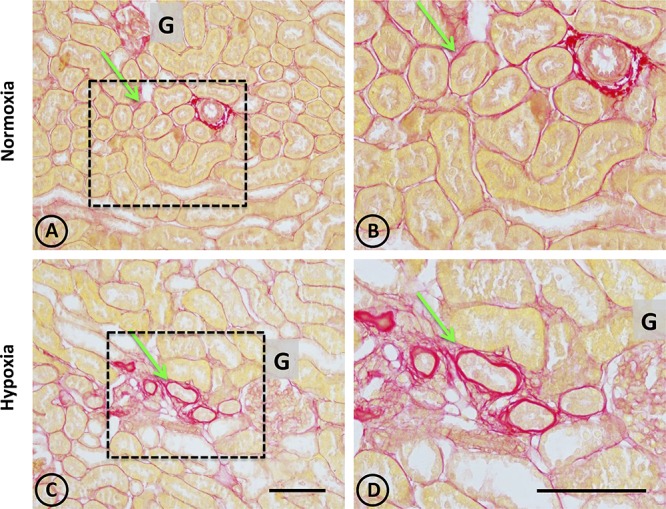
Collagen staining with Picrosirius red stain in renal tissue from normoxic and hypoxic animals. **(A,B)** correspond to the normoxia group. **(C,D)** correspond to the hypoxia group. The left panel shows a panoramic view of the cortical and medullary zones of both groups. The dotted area indicates images with higher magnification on the right column. The arrows indicate the location of collagen stained in red. Note that peritubular collagen is scarce in all images. However, in the hypoxia group it is possible to observe small areas of fibrosis, characterized by an increase in local collagen. In **(A,B)** an arterial blood vessel is observed with perivascular collagen, which does not constitute fibrosis. All structures other than collagen are stained in yellow. G corresponds to the glomerulus. Scale bar = 100 μm.

In normal tissue, staining with Picrosirius red revealed typical collagen staining around arteries, and very faint staining in peritubular locations (Figures 4A,B). On the other hand, focal staining was observed in tissue from hypoxic animals in discrete areas corresponding to focal areas of local injury, as presented in Figures 4C,D, where peritubular signs of fibrosis around dilated and injured tubules were observed.

Thus, the histological evidence presented above is consistent with mild manifestations of lesions, with focal distribution patterns located in the outer medulla and inner cortical zone of the kidneys. No similar lesions were observed in the inner medulla or in the outer cortical zone of normoxic animals.

It is relevant to note that this outer medullary/inner cortical area is the zone where juxtamedullary nephrons reside. Juxtamedullary nephrons are fewer than cortical or superficial nephrons, but are very important for sodium management and blood pressure regulation because they generate medullary circulation.

### Vasoactive Enzyme Expression and Distribution

#### Kallikrein

**FIGURE 5:**
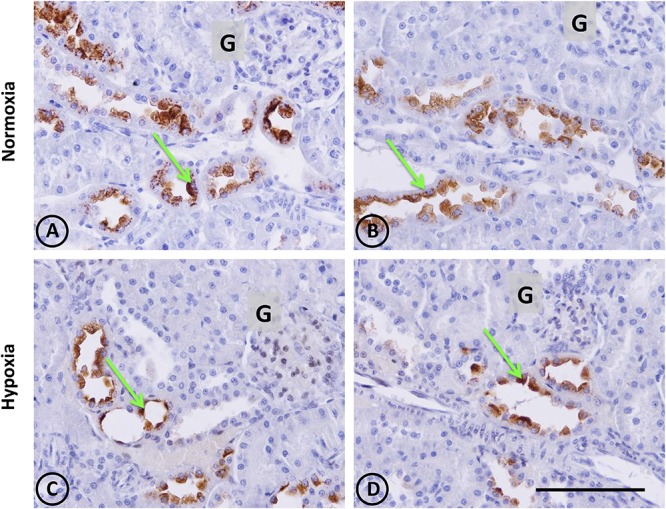
Kallikrein immunostaining in renal tissue from normoxic and hypoxic animals. Green arrows indicate the localization of kallikrein (brown immunostaining) in both groups. It is expressed only in the cortical zone, specifically in the apical pole and perinuclear area of connecting tubule cells. **(A,B)** correspond to the normoxia group. Greater expression of kallikrein stains are observed in this group compared to the hypoxia group **(C,D)**, which presents less staining in the same cell type. The immunostained mark is not present in the collecting duct or another tubule type, in the glomerulus (G), or in blood vessels. All images of the cortical zone have the same magnification. Scale bar = 100 μm.

In normal and hypoxic experimental groups, kallikrein is restricted to connecting tubular cells (CNTc) of CNT of distal nephrons. In normal tissue, CNTc display abundant immunoreactive kallikrein in the apical pole and in the perinuclear area, where the Golgi complex resides. CNTc are intermingled with kallikrein negative cells, which correspond to intercalated cells. CNT are always in close anatomical relation with afferent arterioles close to glomeruli (Figures 5A,B). Kallikrein distribution in renal tissue from hypoxic animals was similar to control animals in the CNTc. However, decreased immunostaining intensity and stained focal areas were observed, and there was faint staining in focal tubules (Figures 5C,D).

**FIGURE 6:**
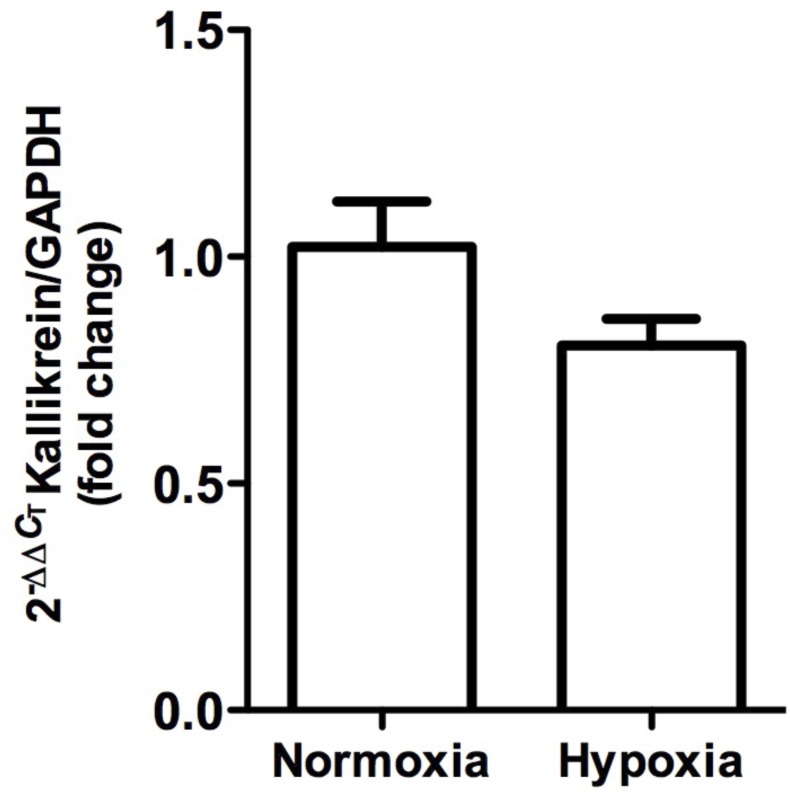
Kallikrein gene expression in whole kidney tissue samples from normoxic and hypoxic animals. The graph represents the standardized average of kallikrein gene expression in both conditions, expressed as fold change between control versus hypoxic group. Results were averaged and mean values were compiled for statistical analysis. A slight decrease is observed, which does not constitute a statistically significant difference between the normoxia and hypoxia groups. GAPDH was used as a housekeeping gene.

Kallikrein gene expression measured by qRT-PCR was not significantly modified (*P* > 0.05), although it tended to decrease (Figure 6).

#### Angiotensin-Converting Enzyme

**FIGURE 7:**
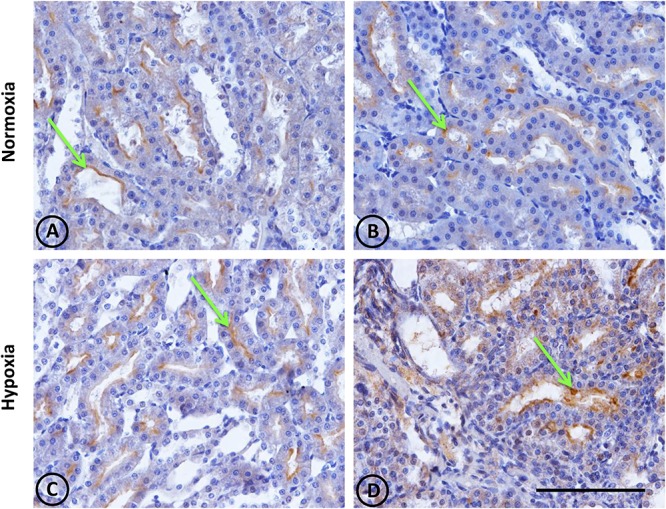
Angiotensin-converting enzyme (ACE) immunostaining in renal tissue from normoxic and hypoxic animals. ACE localization (immunostained in brown) is indicated with green arrows. It is mainly expressed in the medullary zone, at the brush border of the proximal tubule; more specifically in segment S3. **(A,B)** correspond to the normoxia group. The ACE stain is less in the control group compared to the hypoxia group **(C,D)**. Additionally, in image **(D)**, the immunostained mark appears in the tubulointerstitial space, where there is also a higher number of infiltrative cells. All images correspond to the medullary zone with the same magnification. Scale bar = 100 μm.

In normal animals, ACE was located exclusively in the S3 segment of proximal tubules, and was more concentrated in the inner cortical zone and outer medulla. The enzyme was present in the brush border of the apical cellular pole. No ACE was observed in other tubular cells or in the tubulointerstitial space (Figures 7A,B). In hypoxic animals, the same pattern of localization was observed over the proximal tubules, although with more intensity. Furthermore, strong staining in more disarranged proximal tubules was observed in focal areas, and focal ACE staining on the peritubular side (Figures 7C,D).

**FIGURE 8:**
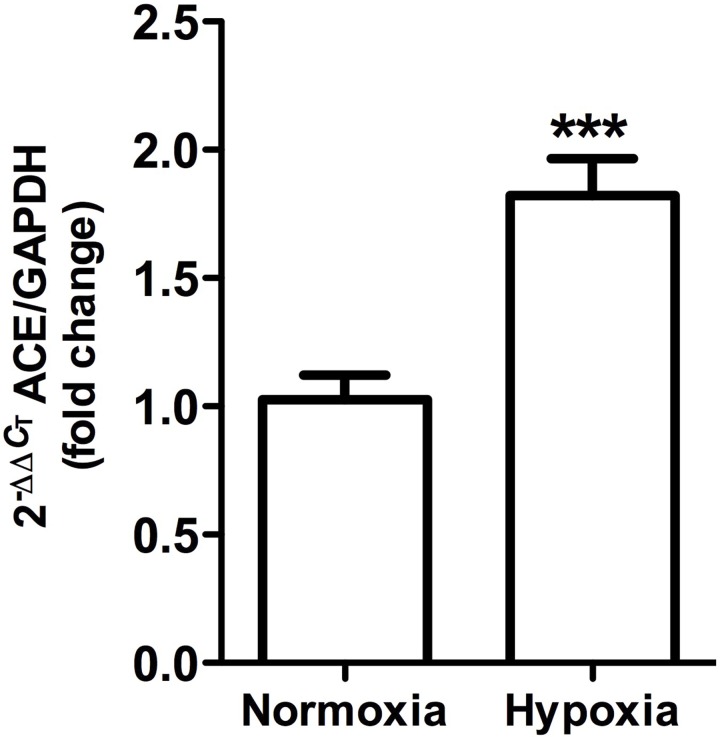
Angiotensin-converting enzyme gene expression in whole kidney tissue samples from normoxic and hypoxic animals. The graph exhibits the average of ACE gene expression. Units were defined as fold change between control versus hypoxic group. Results were averaged and mean values were compiled for statistical analysis. There is a significant increase (^∗∗∗^*P* < 0.001) in ACE gene expression in the hypoxia group when compared to the normoxia group. GAPDH was used as a housekeeping gene.

ACE gene expression, as measured by qRT-PCR, was significantly higher (*P* < 0.001) in hypoxic animals compared to controls (Figure 8).

#### Neutral Endopeptidase 24.11 or Neprylisin

**FIGURE 9:**
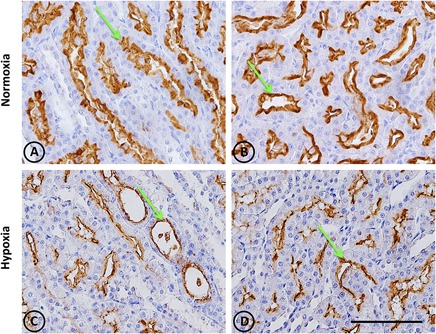
Neutral endopeptidase immunostaining in renal tissue from normoxic and hypoxic animals. The NEP protein is indicated with green arrows, and it appears immunostained in brown. Its expression was located in proximal tubules, being more concentrated in the S3 segment (both in superficial and juxtamedullary nephrons) at the medullary ray and outer strip of the medulla, respectively. **(A,B)** correspond to the normoxia group. NEP expression is higher in the control group compared to the hypoxia group **(C,D)**. All images correspond to the medullary zone, and they have the same magnification. Scale bar = 100 μm.

**FIGURE 10:**
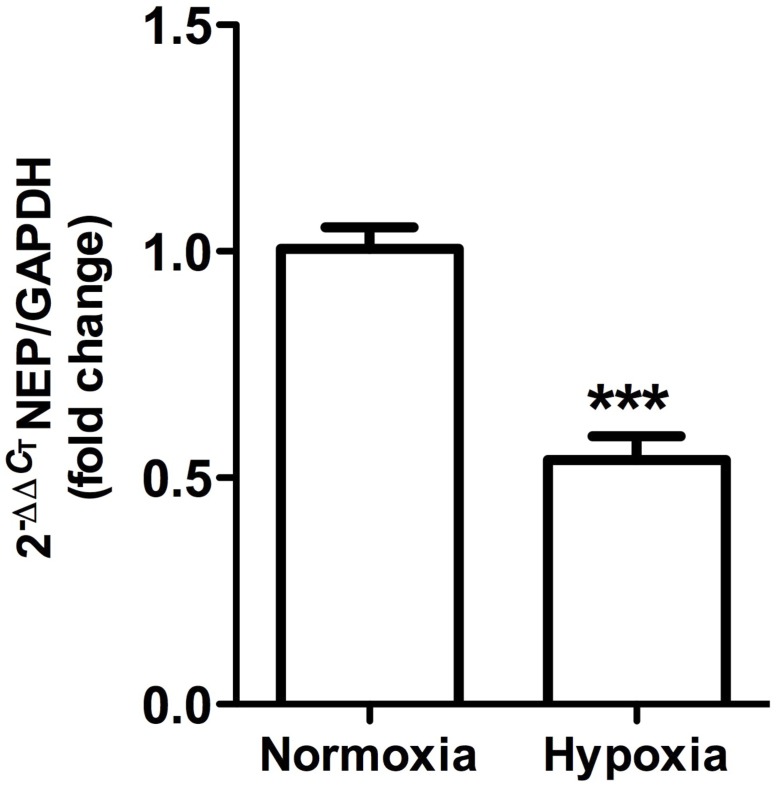
Neutral endopeptidase gene expression in whole kidney tissue samples from normoxic and hypoxic animals. The graph shows the average of NEP gene expression. Arbitrary units were defined as fold change between control versus hypoxic group. Results were averaged and mean values were compiled for statistical analysis. There is a significant decrease in NEP gene expression in the hypoxia group compared to the normoxia group (^∗∗∗^*P* < 0.001). GAPDH was used as a housekeeping gene.

This was observed in control animals in the brush border of proximal tubules, and more concentrated in the outer medulla than in the cortex. Heavy staining was observed in control renal tissue (Figures 9A,B), whereas less area was stained in hypoxic kidneys compared to controls. Furthermore, the presence of NEP in focal areas was observed in rather dilated and atrophied tubules (Figures 9C,D). Moreover, NEP gene expression, as measured by qRT-PCR, significantly decreased (*P* < 0.001) by almost 50% in hypoxic animals compared to controls (Figure 10).

## Discussion

Hypoxia due to renal vasoconstriction has been proposed to contribute to early stages of CKD and salt-sensitive hypertension (Suga et al., 2001; Johnson et al., 2002). The main findings of the present study are that normobaric hypoxia (10% O_2_) for 2 weeks induced subtle renal injury, with discrete focal lesions, and local changes in components of renal vasoactive systems, such as increases in kallikrein and ACE, and less NEP expression.

Normobaric hypoxia may be induced by a variety of conditions that reduce the supply of oxygen to organs, such as chronic obstructive pulmonary disease (Kent et al., 2011), obstructive sleep apnea syndrome (Chiang, 2006), CKD (Shoji et al., 2014), and certain conditions that induce local renal hypoxia like cyclosporine A, hypokalemia, angiotensin II or contrast media (Lombardi et al., 2001; Suga et al., 2001; Zhou and Duan, 2014; Fähling et al., 2017). Normobaric hypoxia studies have an advantage over experiments using hypobaric conditions in that hypoxia effects are not mixed with those of low pressure. In fact, arterial blood pressure has been reported to remain unchanged in chronic hypobaric hypoxia (Rabinovitch et al., 1979; Hsieh et al., 2015), but to increase in chronic normobaric hypoxia (Schwenke et al., 2008; Moraes et al., 2014; Flor et al., 2018). The latter highlights the importance of choosing the right type of hypoxia depending on the pathological conditions to be studied, since both conditions may evoke distinct physiological responses (Savourey et al., 2003; Richard and Koehle, 2012). Hence, we used the normobaric hypoxia model in order to mimic the low oxygen supply that kidneys face under pathological conditions, and to distinguish the effects of hypoxia from hypobaria.

### Morphological Alterations

Hypoxia is a key pathogenic event that can activate a vicious cycle of destructive processes. In our model, we observed a discrete focal lesion of tubular damage with conventional H-E, and Picrosirius red staining without glomerular damage, indicating the mild and patchy nature of the lesion. The expression of tubular OPN, which is a key macrophage chemokine, without infiltrative cells was somehow unexpected. However, it suggests that longer periods or more severe hypoxia are required to induce infiltration. When compared to other models of mild damage (angiotensin II, hypokalemia, phenylephrine and cyclosporine A) (Johnson et al., 2002), the present model displays a similar damage pattern, but with less intensity. A possible explanation for the low intensity of the damage is that this degree of hypoxia is counteracted in kidneys by regulatory mechanisms that supply oxygen to kidneys as the increased hematocrit (Table 1). Similar results have been shown in proximal and distal tubular cells in renal ischemia, tubulointerstitial nephritis, glomerulonephritis, and acute hypoxia (Hampel et al., 2003; Mazzali et al., 2003). These effects are in agreement with upregulation of inflammatory and profibrotic genes in response to hypoxia (Fu et al., 2016; Ow et al., 2018). Fibrosis and subsequent tubular damage exacerbate hypoxia, and induce the activation of genes that favor the expression of vasoconstrictor mediators such as endothelin-1, which could reduce oxygen delivery even more (Hampel et al., 2003). Considering that the outer medulla of the kidney is particularly vulnerable to reduced oxygen supply due to an imbalance between oxygen requirements and medullary blood flow (Fine et al., 2000; Zhou and Duan, 2014), we and others have proposed that a common pathway is perhaps local hypoxia, which creates a vicious circle that initiates and maintains focal renal injury (Vio and Jeanneret, 2003).

A larger inflammatory response was described in rats subjected to chronic hypoxia (81.9 mmHg) (Mazzali et al., 2003). The latter study achieved low PO_2_ by reducing barometric pressure to an equivalent altitude of 5550 m, although PO_2_ reduction was more severe in the present study (67.1 mmHg). This reduction in barometric pressure, however, may induce physiological responses that are independent from PO_2_ reduction (Savourey et al., 2003, 2007; Richard and Koehle, 2012). Moreover, the exposure period was 10 days shorter in the experiments reported here. In chronic intermittent hypoxic experiments, changes in arterial pressure did not develop until 3 weeks of exposure (Del Rio et al., 2010). Thus, differences may arise from the different exposure period and barometric pressure.

### Alterations in Vasoactive Systems

Changes in renal vasoactive components have been proposed to contribute to early stages of CKD and salt-sensitive hypertension. In fact, Johnson et al. (2002) proposed that changes in vasoactive systems constitute a common base for a variety of stimuli (e.g., hypokalemia, angiotensin II, phenylephrine, cyclosporine A) that induce vasoconstriction, leading to ischemia and subsequent subtle renal injury. In such conditions, rats are more sensitive to high-salt induced hypertension, even though arterial blood pressure augmentation is reversed after removing the stimuli. This suggests that renal damage is at the base of this mechanism.

### Decreased Kallikrein Levels

Kallikrein is a serine protease with a key function for kidneys, since it produces bradykinin, which is one of the main vasorelaxing peptides that also inhibits sodium reabsorption through the activation of bradykinin receptor type 2 (Mukai et al., 1996; Sivritas et al., 2008). Our histology and immunohistochemistry data showed a local decrease of kallikrein in CNTc. It is worth noting that kallikrein-containing cells are always in close contact with arterioles underlying the anatomical link between KKS and RAS (Vio et al., 1988). Even though changes in kallikrein gene expression were not significant, there was a tendency to decrease. We have previously reported that hypokalemia, ischemia and phenylephrine (stimuli that produce renal hypoxia and kidney damage) also reduced kallikrein expression, which could contribute to the imbalance of vasoactive enzymes that favor vasoconstriction and sodium retention. The latter may, in turn, lead to the onset of renal injury and hypertension (Martinez et al., 1990; Suga et al., 2001; Johnson et al., 2002; Basile et al., 2005). Further studies are required to determine whether kallikrein reduction is a cause or a consequence of hypoxia. Thongboonkerd et al. (2002) reported a dual role of kallikrein, where episodic hypoxia (which mimics obstructive sleep apnea syndrome) reduced its activity associated with hypertension, while sustained hypoxia increased kallikrein levels. The difference in kallikrein expression observed under the experimental conditions reported here may have been due to the reoxygenation produced between the end of hypoxia and the extraction of renal tissue. This could be explained by the oxidative stress generated during reoxygenation, which may activate different pathways, and could influence kallikrein expression (Milano et al., 2004; Bianciardi et al., 2006). Reduced kallikrein expression favors vasoconstriction, which may worsen hypoxia and lead to kidney damage, since overexpression of human kallikrein in kidneys has been observed to protect rats from hypoxia-induced hypertension (Thongboonkerd et al., 2002).

### Increased ACE Levels

ACE plays a critical role in RAS signaling: It produces angiotensin II (the main vasoconstrictor peptide), which activates pathways leading to hypertension and fibrosis, while also degrading bradykinin. Results from immunohistochemistry in this study showed a local increase of ACE in proximal tubules at the outer medullary region. The observed change in protein expression is consistent with greater gene expression, as measured by qRT-PCR. For many years, much effort has been placed on creating drugs to inhibit ACE, but very little is known about its regulation. It has been reported that phenylephrine, angiotensin II infusion and hypokalemia induce the ACE expression, which has been associated with kidney hypoxia and damage (Johnson et al., 1999; Lombardi et al., 2001; Suga et al., 2001; Vio and Jeanneret, 2003).

It seems that ACE regulation differs depending on the tissue analyzed (Jackson et al., 1986; Oparil et al., 1988; King et al., 1989). For instance, Oparil et al. (1988) reported that hypoxia reduced ACE activity in lungs (associated to angiotensin II reduction), but increased its activity in renal tissue, which was reversed to normal levels when rats were returned to normoxia. Reasons behind organ-specific regulation of ACE are not known, but lungs and kidneys seem to present opposite ACE regulations. Vascular endothelial growth factor (VEGF) is postulated to be the major growth factor modified in response to hypoxia, which increases local production of ACE (Enholm et al., 1997; Saijonmaa et al., 2001), and generates synergy between the RAS and VEGF, hence contributing to angiogenesis and vascular remodeling in response to hypoxia.

Increases in ACE may be acting in the following two ways: (1) to increase the amount of angiotensin II that leads to greater vascular resistance and fibrosis, and (2) to decrease the amount of kallikrein by degradation, contributing to an imbalance of vasoactive enzymes and thus to the onset of renal injury observed during hypoxia.

### Decreased NEP Levels

Together with ACE, NEP is the most important kinin-degrading enzyme in the cardiovascular system. Renal NEP is responsible for processing a range of substrates as vasoactive peptides, including bradykinin, endothelins, angiotensin I and angiotensin II, among others (Rice et al., 2004; Judge et al., 2015; Campbell, 2017). In our model, we observed that hypoxia decreased NEP expression both at protein and gene expression levels. Immunohistochemical staining showed that NEP is abundantly expressed in proximal tubules at the outer medullary region under normoxic conditions, but hypoxia reduced its expression. One of the consequences of NEP reduction is a lower generation of angiotensin 1–7, an antihypertensive peptide that counteracts the effects of angiotensin II, which is consistent with a hypertensive phenotype.

There are few studies that report on NEP localization, although the enzyme exhibits vast tissue distribution, with greater abundance in kidneys and lungs (Li et al., 1995). Previous studies by Carpenter and Stenmark (2001) showed that normobaric hypoxia, although for a much shorter time (72 h), caused a decline in NEP expression at the pulmonary level, which caused increases in vascular permeability. The latter corresponds to a phenotype that is reversed through recombinant NEP administration or a bradykinin receptor antagonist. Down-regulation of NEP by hypoxia has been reported in mouse primary cortical and hippocampal neurons as well as in prostate cancer cell lines, where NEP decrease is associated with a loss of beneficial effects mediated by the specific degradation of the substrate (Wang et al., 2011; Nalivaeva et al., 2012; Mitra et al., 2013). It has been shown that the hypoxia inducible factor negatively regulates NEP expression, due to the presence of hypoxia-responsive elements in its promoter (Mitra et al., 2013), which could be responsible for NEP reduction.

Taken together, the changes in the enzymes that regulate the production/degradation of the vasoactive peptides observed in our experimental conditions suggest that hypoxia induces a hypertensive phenotype due to an imbalance of the vasoactive peptides, leading to an increase in the vasoconstrictors and a decrease in vasodilators, such as: decreased bradykinin (antihypertensive peptide) due to decreased kallikrein and increased ACE; increased angiotensin II (hypertensive peptide) due to increased ACE; and reduction of angiotensin 1–7 (antihypertensive peptides) due to decreased NEP.

In summary, we describe local and subtle renal injuries and changes in renal vasoactive components that could damage the kidney, making it more sensitive to high-salt induced hypertension under conditions such as high salt load. Future experiments could involve challenging renal function with high sodium diets to increase oxygen consumption and induce salt-sensitive hypertension.

## Study Limitations

Although this study attempted to limit oxygenation time before collecting the samples (2 h maximum) and during feeding/cleaning the animals (5 min every 48 h), changes occurring during reoxygenation (e.g., changes in redox status) could not be ruled out, and may have altered our results. Additionally, renal function could not be measured, since the metabolic cages could not be placed under hypoxic conditions.

Due to space limitations in the hypoxic chambers, the experiments were performed using only male rats. Considering the important differences between males and females in terms of cardiovascular and renal function, it is important to perform these experiments on females in the future to evaluate the possible differences that may exist in the response to hypoxia.

Finally, changes in the expression of key enzymes of the RAS and the KKS were measured, but it cannot be assumed that this will translate into changes in peptide levels. Local levels of angiotensin II and bradykinin will be measured and evaluated in future studies.

## Author Contributions

CV, JA, RI, and CC conceived and designed the study, analyzed and interpreted the data, drafted the manuscript, critically revised important intellectual content in the manuscript, and provided overall supervision. DS, CC, JA, and JD-E performed the experiments and analyzed the data and drafted the manuscript, and contributed to intellectual content in the manuscript. NM analyzed and interpreted the data and contributed to intellectual content in the manuscript. All authors approved the final manuscripts and agreed to be accountable for all aspects of the work.

## Conflict of Interest Statement

The authors declare that the research was conducted in the absence of any commercial or financial relationships that could be construed as a potential conflict of interest.

## References

[B1] Bader M. Ganten D. (2008). Update on tissue renin-angiotensin systems. *J. Mol. Med.* 86 615–621. 10.1007/s00109-008-0336-0 18414822

[B2] Basile D. P. Fredrich K. Alausa M. Vio C. P. Liang M. Rieder M. R. (2005). Identification of persistently altered gene expression in the kidney after functional recovery from ischemic acute renal failure. *Am. J. Physiol. Renal Physiol.* 288 F953–F963. 10.1152/ajprenal.00329.2004 15632414

[B3] Baumgärtl H. Leichtweiss H. P. Lübbers D. W. Weiss C. Hurland H. (1972). The oxygen supply of the dog kidney: measurements of intrarenal pO2. *Microvasc. Res.* 4 247–257. 10.1016/0026-2862(72)90036-2 5043917

[B4] Bianciardi P. Fantacci M. Caretti A. Ronchi R. Milano G. Morel S. (2006). Chronic in vivo hypoxia in various organs: hypoxia-inducible factor-1alpha and apoptosis. *Biochem. Biophys. Res. Commun.* 342 875–880. 10.1016/j.bbrc.2006.02.042 16596722

[B5] Campbell D. J. (2017). Long-term neprilysin inhibition - implications for ARNIs. *Nat. Rev. Cardiol.* 14 171–186. 10.1038/nrcardio.2016.200 27974807

[B6] Carpenter T. C. Stenmark K. R. (2001). Hypoxia decreases lung neprilysin expression and increases pulmonary vascular leak. *Am. J. Physiol. Lung Cell. Mol. Physiol.* 281 L941–L948. 10.1152/ajplung.2001.281.4.L941 11557598

[B7] Chen Y. F. (2005). Atrial natriuretic peptide in hypoxia. *Peptides* 26 1068–1077. 10.1016/j.peptides.2004.08.030 15911074

[B8] Chiang A. A. (2006). Obstructive sleep apnea and chronic intermittent hypoxia: a review. *Chin. J. Physiol.* 249 234–243. 17294831

[B9] Del Rio R. Moya E. A. Iturriaga R. (2010). Carotid body and cardiorespiratory alterations in intermittent hypoxia: the oxidative link. *Eur. Respir. J.* 36 136–142. 10.1183/09031936.00158109 19996187

[B10] Enholm B. Paavonen K. Ristimäki A. Kumar V. Gunji Y. Klefstrom J. (1997). Comparison of VEGF, VEGF-B, VEGF-C and Ang-1 mRNA regulation by serum, growth factors, oncoproteins and hypoxia. *Oncogene* 14 2475–2483. 10.1038/sj.onc.1201090 9188862

[B11] Epstein F. H. (1997). Oxygen and renal metabolism. *Kidney Int.* 51 381–385. 10.1038/ki.1997.50 9027710

[B12] Fähling M. Mathia S. Scheidl J. Abramovitch R. Milman Z. Paliege A. (2017). Cyclosporin a induces renal episodic hypoxia. *Acta Physiol.* 219 625–639. 10.1111/apha.12811 27690155

[B13] Fine L. G. Bandyopadhay D. Norman J. T. (2000). Is there a common mechanism for the progression of different types of renal diseases other than proteinuria? Towards the unifying theme of chronic hypoxia. *Kidney Int. Suppl.* 75 S22–S26. 10.1046/j.1523-1755.2000.07512.x 10828757

[B14] Flor K. C. Silva E. F. Menezes M. F. Pedrino G. R. Colombari E. Zoccal D. B. (2018). Short-term sustained hypoxia elevates basal and hypoxia-induced ventilation but not the carotid body chemoreceptor activity in rats. *Front. Physiol.* 9:134. 10.3389/fphys.2018.00134 29535636 PMC5835044

[B15] Fu Q. Colgan S. P. Shelley C. S. (2016). Hypoxia: the force that drives chronic kidney disease. *Clin. Med. Res.* 14 15–39. 10.3121/cmr.2015.1282 26847481 PMC4851450

[B16] Gess B. Schricker K. Pfeifer M. Kurtz A. (1997). Acute hypoxia upregulates NOS gene expression in rats. *Am. J. Physiol. Regul. Integr. Comp. Physiol.* 273 R905–R910. 10.1152/ajpregu.1997.273.3.R905 9321866

[B17] Günther H. Aumüller G. Kunke S. Vaupel P. Thews G. (1974). Verteilung der O2-drucke in der rattenniere unter normbedingungen. *Res. Exp. Med.* 163 251–264. 10.1007/BF01851672 4603896

[B18] Hampel D. J. Sansome C. Romanov V. I. Kowalski A. J. Denhardt D. T. Goligorsky M. S. (2003). Osteopontin traffic in hypoxic renal epithelial cells. *Nephron Exp. Nephrol.* 94 e66–e76. 10.1159/000071285 12845232

[B19] Hargrove G. M. Dufresne J. Whiteside C. Muruve D. A. Wong N. C. (2000). Diabetes mellitus increases endothelin-1 gene transcription in rat kidney. *Kidney Int.* 58 1534–1545. 10.1046/j.1523-1755.2000.00315.x 11012888

[B20] Hsieh Y. H. Jacono F. J. Siegel R. E. Dick T. E. (2015). Respiratory modulation of sympathetic activity is attenuated in adult rats conditioned with chronic hypobaric hypoxia. *Respir. Physiol. Neurobiol.* 206 53–60. 10.1016/j.resp.2014.11.011 25462835 PMC4314614

[B21] Icekson G. Dominguez C. V. Dedios V. P. Arroyo J. Alcayaga J. (2013). Petrosal ganglion responses to acetylcholine and ATP are enhanced by chronic normobaric hypoxia in the rabbit. *Respir. Physiol. Neurobiol.* 189 624–631. 10.1016/j.resp.2013.07.023 23969181

[B22] Jackson R. M. Narkates A. J. Oparil S. (1986). Impaired pulmonary conversion of angiotensin I to angiotensin II in rats exposed to chronic hypoxia. *J. Appl. Physiol.* 60 1121–1127. 10.1152/jappl.1986.60.4.1121 3009385

[B23] Jaffa A. A. Vio C. P. Silva R. H. Vavrek R. J. Stewart J. M. Rust P. F. (1992). Evidence for renal kinins as mediators of amino acid-induced hyperperfusion and hyperfiltration in the rat. *J. Clin. Invest* 89 1460–1468. 10.1172/JCI115736 1373739 PMC443016

[B24] Johnson R. J. Gordon K. L. Suga S. Duijvestijn A. M. Griffin K. Bidani A. (1999). Renal injury and salt-sensitive hypertension after exposure to catecholamines. *Hypertension* 34 151–159. 10.1161/01.HYP.34.1.151 10406839

[B25] Johnson R. J. Herrera-Acosta J. Schreiner G. F. Rodriguez-Iturbe B. (2002). Subtle acquired renal injury as a mechanism of salt-sensitive hypertension. *N. Engl. J. Med.* 346 913–923. 10.1056/NEJMra011078 11907292

[B26] Judge P. Haynes R. Landray M. J. Baigent C. (2015). Neprilysin inhibition in chronic kidney disease. *Nephrol. Dial. Transplant.* 30 738–743. 10.1093/ndt/gfu269 25140014 PMC4425478

[B27] Kelsen S. Hall J. E. Chade A. R. (2011). Endothelin-A receptor blockade slows the progression of renal injury in experimental renovascular disease. *Am. J. Physiol. Renal Physiol.* 301 F218–F225. 10.1152/ajprenal.00089.2011 21478482 PMC3129888

[B28] Kent B. D. Mitchell P. D. McNicholas W. T. (2011). Hypoxemia in patients with COPD: cause, effects, and disease progression. *Int. J. Chron. Obstruct. Pulmon. Dis.* 6 199–208. 10.2147/COPD.S10611 21660297 PMC3107696

[B29] King S. J. Booyse F. M. Lin P. H. Traylor M. Narkates A. J. Oparil S. (1989). Hypoxia stimulates endothelial cell angiotensin-converting enzyme antigen synthesis. *Am. J. Physiol. Cell Physiol.* 256 C1231–C1238. 10.1152/ajpcell.1989.256.6.C1231 2544094

[B30] Leichtweiss H. P. Lübbers D. W. Weiss C. Baumgärtl H. Reschke W. (1969). The oxygen supply of the rat kidneys: measurements of intrarenal pO2. *Pflügers Arch.* 309 328–349. 10.1007/BF00587756 5816079

[B31] Li C. Booze R. M. Hersh L. B. (1995). Tissue-specific expression of rat neutral endopeptidase (neprilysin) mRNAs. *J. Biol. Chem.* 270 5723–5728. 10.1074/jbc.270.11.5723 7890699

[B32] Livak K. J. Schmittgen T. D. (2001). Analysis of relative gene expression data using real-time quantitative PCR and the 2^-ΔΔCT^ method. *Methods* 25 402–408. 10.1006/meth.2001.1262 11846609

[B33] Lombardi D. M. Viswanathan M. Vio C. P. Saavedra J. M. Schwartz S. M. Johnson R. J. (2001). Renal and vascular injury induced by exogenous angiotensin II is AT1 receptor-dependent. *Nephron* 87 66–74. 10.1159/000045886 11174028

[B34] Lübbers D. W. Baumgärtl H. (1997). Heterogeneities and profiles of oxygen pressure in brain and kidney examples of pO2 distribution in the living tissue. *Kidney Int.* 51 372–380. 10.1038/ki.1997.49 9027709

[B35] Madeddu P. Emanueli C. El-Dahr S. (2007). Mechanisms of disease: the tissue kallikrein-kinin system in hypertension and vascular remodeling. *Nat. Clin. Pract. Nephrol.* 3 208–221. 10.1038/ncpneph0444 17389890

[B36] Martinez L. Vio C. P. Valdés G. Kychenthal W. Mendoza S. (1990). Decreased kallikrein excretion in renal transplant treated with cyclosporine A. *Transplant. Proc.* 22 294–296. 2309333

[B37] Mazzali M. Jefferson J. A. Ni Z. Vaziri N. D. Johnson R. J. (2003). Microvascular and tubulointerstitial injury associated with chronic hypoxia-induced hypertension. *Kidney Int.* 63 2088–2093. 10.1046/j.1523-1755.2003.00011.x 12753295

[B38] Meyers K. E. Sethna C. (2013). Endothelin antagonists in hypertension and kidney disease. *Pediatr. Nephrol.* 28 711–720. 10.1007/s00467-012-2316-4 23070275

[B39] Milano G. Bianciardi P. Corno A. F. Raddatz E. Morel S. von Segesser L. K. (2004). Myocardial impairment in chronic hypoxia is abolished by short aeration episodes: involvement of K+ATP channels. *Exp. Biol. Med.* 229 1196–1205. 10.1177/153537020422901115 15564447

[B40] Mitra R. Chao O. S. Nanus D. M. Goodman O. B. Jr. (2013). Negative regulation of NEP expression by hypoxia. *Prostate* 73 706–714. 10.1002/pros.22613 23138928

[B41] Mizutani S. Ishii M. Hattori A. Nomura S. Numaguchi Y. Tsujimoto M. (2008). New insights into the importance of aminopeptidase A in hypertension. *Heart Fail. Rev.* 13 273–284. 10.1007/s10741-007-9065-7 17990103 PMC7101674

[B42] Moraes D. J. A. Bonagamba L. G. A. Costa K. M. Costa-Silva J. H. Zoccal D. B. Machado B. H. (2014). Short-term sustained hypoxia induces changes in the coupling of sympathetic and respiratory activities in rats. *J. Physiol.* 592 2013–2033. 10.1113/jphysiol.2013.262212 24614747 PMC4230776

[B43] Mukai H. Fitzgibbon W. R. Bozeman G. Margolius H. S. Ploth D. W. (1996). Bradykinin B2 receptor antagonist increases chloride and water absorption in rat medullary collecting duct. *Am. J. Physiol. Regul. Integr. Comp. Physiol.* 271 R352–R360. 10.1152/ajpregu.1996.271.2.R352 8770134

[B44] Nalivaeva N. N. Belyaev N. D. Zhuravin I. A. Turner A. J. (2012). The Alzheimer’s amyloid-degrading peptidase, neprilysin: can we control it? *Int. J. Alzheimers Dis.* 2012:383796. 10.1155/2012/383796 22900228 PMC3412116

[B45] Nangaku M. Fujita T. (2008). Activation of the renin-angiotensin system and chronic hypoxia of the kidney. *Hypertens. Res.* 31 175–184. 10.1291/hypres.31.175 18360035

[B46] O’Neill J. Fasching A. Pihl L. Patinha D. Franzén S. Palm F. (2015). Acute SGLT inhibition normalizes O2 tension in the renal cortex but causes hypoxia in the renal medulla in anaesthetized control and diabetic rats. *Am. J. Physiol. Renal Physiol.* 309 F227–F234. 10.1152/ajprenal.00689.2014 26041448

[B47] Oparil S. Narkates A. J. Jackson R. M. Ann H. S. (1988). Altered-angiotensin converting enzyme in lung and extrapulmonary tissues of hypoxia-adapted rats. *J. Appl. Physiol.* 65 218–227. 10.1152/jappl.1988.65.1.218 2841276

[B48] Ow C. P. C. Ngo J. P. Ullah M. M. Hilliard L. M. Evans R. G. (2018). Renal hypoxia in kidney disease: cause or consequence? *Acta Physiol.* 222:e12999. 10.1111/apha.12999 29159875

[B49] Rabinovitch M. Gamble W. Nadas A. S. Miettinen O. S. Reid L. (1979). Rat pulmonary circulation after chronic hypoxia: hemodynamic and structural features. *Am. J. Physiol. Heart Circ. Physiol.* 236 H818–H827. 10.1152/ajpheart.1979.236.6.H818 443445

[B50] Rice G. I. Thomas D. A. Grant P. J. Turner A. J. Hooper N. M. (2004). Evaluation of angiotensin-converting enzyme (ACE), its homologue ACE2 and neprilysin in angiotensin peptide metabolism. *Biochem. J.* 383 45–51. 10.1042/BJ20040634 15283675 PMC1134042

[B51] Richard N. A. Koehle M. S. (2012). Differences in cardio-ventilatory responses to hypobaric and normobaric hypoxia: a review. *Aviat. Space Environ. Med.* 83 677–684. 10.3357/ASEM.3182.2012 22779311

[B52] Rodebaugh J. Sekulic M. Davies W. Montgomery S. Khraibi A. Solhaug M. J. (2012). Neuronal nitric oxide synthase, nNOS, regulates renal hemodynamics in the postnatal developing piglet. *Pediatr. Res.* 71 144–149. 10.1038/pr.2011.23 22258124

[B53] Saijonmaa O. Nyman T. Kosonen R. Fyhrquist F. (2001). Upregulation of angiotensin-converting enzyme by vascular endothelial growth factor. *Am. J. Physiol. Heart Circ. Physiol.* 280 H885–H891. 10.1152/ajpheart.2001.280.2.H885 11158990

[B54] Salas S. P. Giacaman A. Romero W. Downey P. Aranda E. Mezzano D. (2007). Pregnant rats treated with a serotonin precursor have reduced fetal weight and lower plasma volume and kallikrein levels. *Hypertension* 50 773–779. 10.1161/HYPERTENSIONAHA.107.094540 17646571

[B55] Savourey G. Launay J. C. Besnard Y. Guinet A. Travers S. (2003). Normo- and hypobaric hypoxia: are there any physiological differences? *Eur. J. Appl. Physiol.* 89 122–126. 10.1007/s00421-002-0789-8 12665974

[B56] Savourey G. Launay J. C. Besnard Y. Guinet-Lebreton A. Alonso A. Sauvet F. (2007). Normo or hypobaric hypoxia tests: propositions for the determination of the individual susceptibility to altitude illnesses. *Eur. J. Appl. Physiol.* 100 193–205. 10.1007/s00421-007-0417-8 17323073

[B57] Schwenke D. O. Tokudome T. Shirai M. Hosoda H. Horio T. Kishimoto I. (2008). Exogenous ghrelin attenuates the progression of chronic hypoxia-induced pulmonary hypertension in conscious rats. *Endocrinology* 149 237–244. 10.1210/en.2007-0833 17916633

[B58] Shoji K. Tanaka T. Nangaku M. (2014). Role of hypoxia in progressive chronic kidney disease and implications for therapy. *Curr. Opin. Nephrol. Hypertens.* 23 161–168. 10.1097/01.mnh.0000441049.98664.6c 24378776

[B59] Sivritas S. H. Ploth D. W. Fitzgibbon W. R. (2008). Blockade of renal medullary bradykinin B2 receptors increases tubular sodium reabsorption in rats fed a normal-salt diet. *Am. J. Physiol. Renal Physiol.* 295 F811–F817. 10.1152/ajprenal.90225.2008 18632797 PMC2536883

[B60] Suga I. Phillips I. Ray P. E. Raleigh J. A. Vio C. P. Kim Y. G. (2001). Hypokalemia induces renal injury and alterations in vasoactive mediators that favor salt sensitivity. *Am. J. Physiol. Renal Physiol.* 281 F620–F629. 10.1152/ajprenal.2001.281.4.F620 11553508

[B61] Thongboonkerd V. Gozal E. Sachleben L. R. Jr. Arthur J. M. Pierce W. M. Cai J. (2002). Proteomic analysis reveals alterations in the renal kallikrein pathway during hypoxia-induced hypertension. *J. Biol. Chem.* 277 34708–34716. 10.1074/jbc.M203799200 12121987

[B62] Villanueva S. Cespedes C. Vio C. P. (2006). Ischemic acute renal failure induces the expression of a wide range of nephrogenic proteins. *Am. J. Physiol. Regul. Integ. Comp. Phys.* 290 R861–R870. 10.1152/ajpregu.00384.2005 16284088

[B63] Villanueva S. Contreras F. Tapia A. Carreño J. E. Vergara C. Ewertz E. (2014). Basic fibroblast growth factor reduces functional and structural damage in chronic kidney disease. *Am. J. Physiol. Renal Physiol.* 306 F430–F441. 10.1152/ajprenal.00720.2012 24285501

[B64] Vio C. P. Figueroa C. D. Caorsi I. (1988). Anatomical relationship between kallikrein containing tubules and the juxtaglomerular apparatus in the human kidney. *Am. J. Hypertens.* 1 269–271. 10.1093/ajh/1.3.269 3390319

[B65] Vio C. P. Jeanneret V. A. (2003). Local induction of angiotensin-converting enzyme in the kidney as a mechanism of progressive renal diseases. *Kidney Int. Suppl.* 86 S57–S63. 10.1046/j.1523-1755.64.s86.11.x 12969129

[B66] Vio C. P. Loyola S. Velarde V. (1992). Localization of components of the kallikrein-kinin system in the kidney: relation to renal function State of the art lecture. *Hypertension* 19 10–16. 10.1161/01.HYP.19.2_Suppl.II10 1735562

[B67] Volpe M. Carnovali M. Mastromarino V. (2016). The natriuretic peptides system in the pathophysiology of heart failure: from molecular basis to treatment. *Clin. Sci.* 130 57–77. 10.1042/CS20150469 26637405 PMC5233571

[B68] Wang Z. Yang D. Zhang X. Li T. Li J. Tang Y. (2011). Hypoxia-induced down-regulation of neprilysin by histone modification in mouse primary cortical and hippocampal neurons. *PLoS One* 6:e19229. 10.1371/journal.pone.0019229 21559427 PMC3084787

[B69] Zhang D. D. Gao Z. X. Vio C. P. Xiao Y. Wu P. Zhang H. (2018). Bradykinin stimulates renal Na+ and K+ excretion by inhibiting the K+ channel (Kir4.1) in the distal convoluted tubule. *Hypertension* 72 361–369. 10.1161/HYPERTENSIONAHA.118.11070 29915013 PMC6043363

[B70] Zhou L. Duan S. (2014). Effects of angiotensin converting enzyme inhibitors and angiotensin receptor blockers in contrast-induced nephropathy. *Kidney Blood Press. Res.* 38 165–171. 10.1159/00035576 24686005

